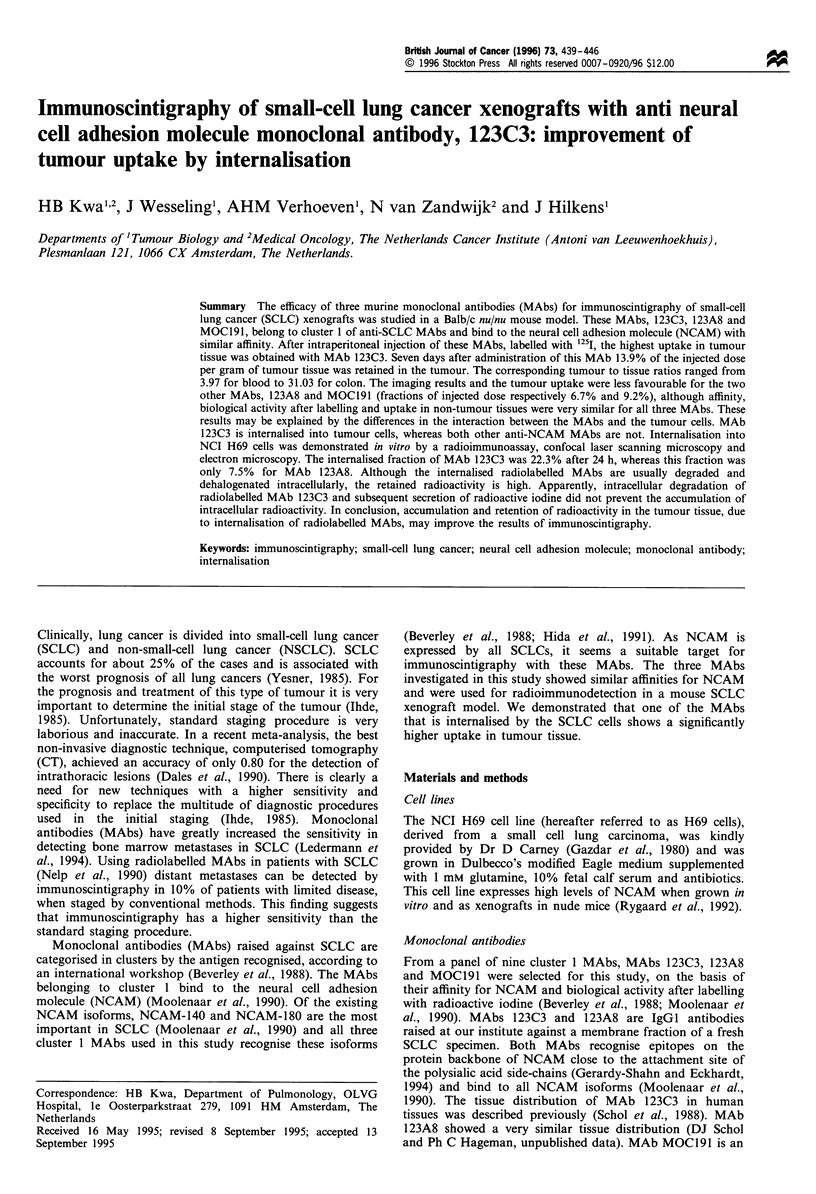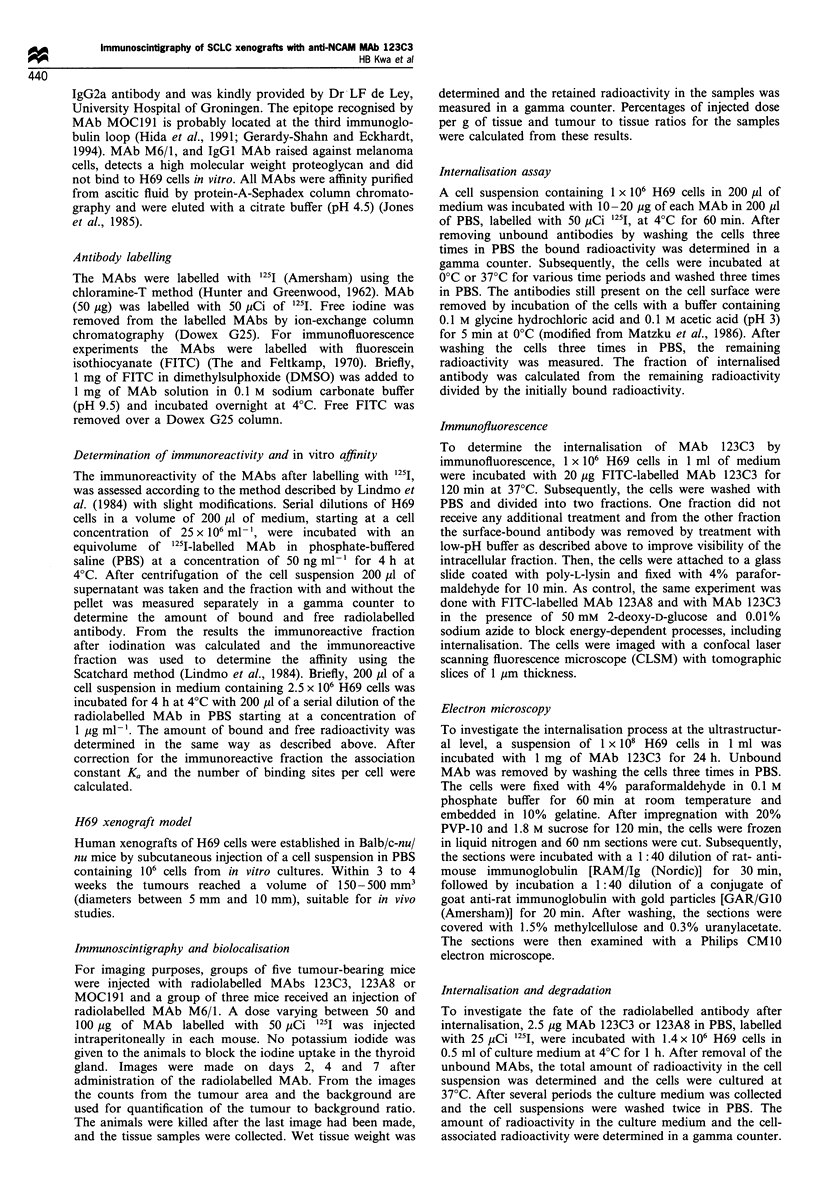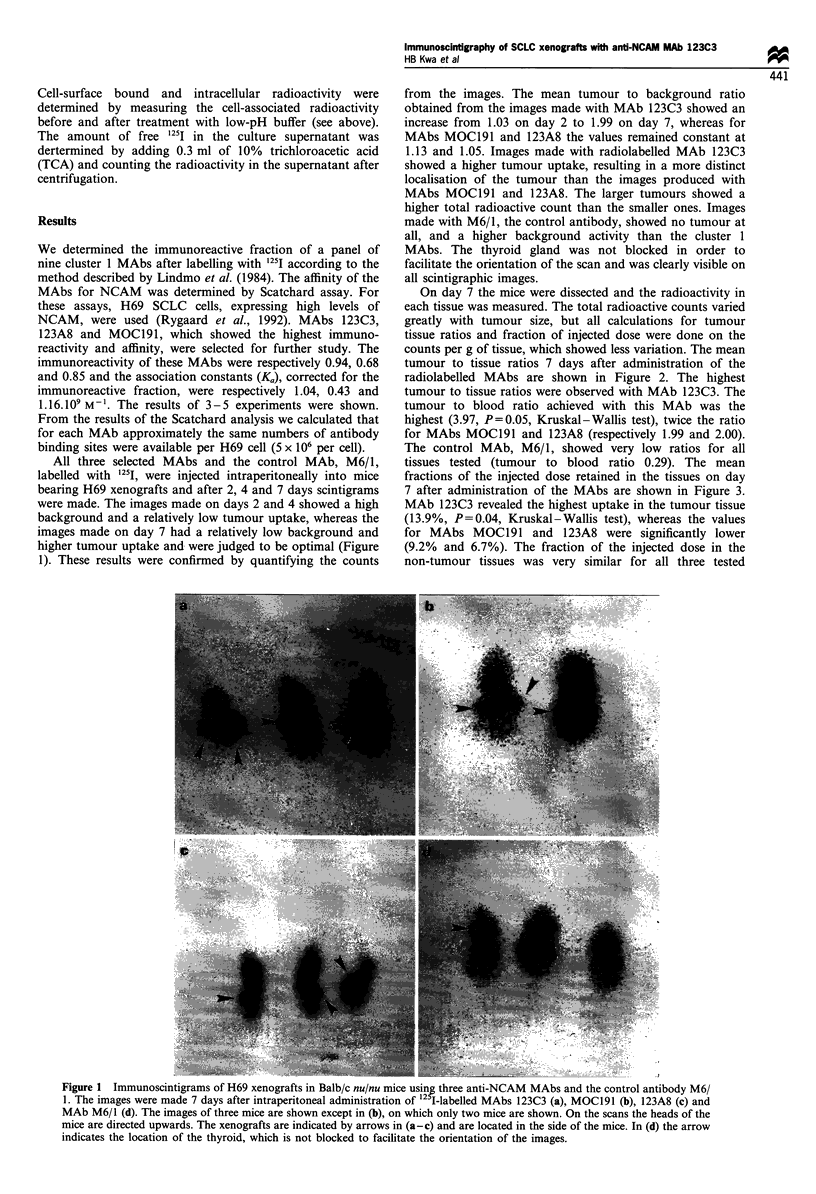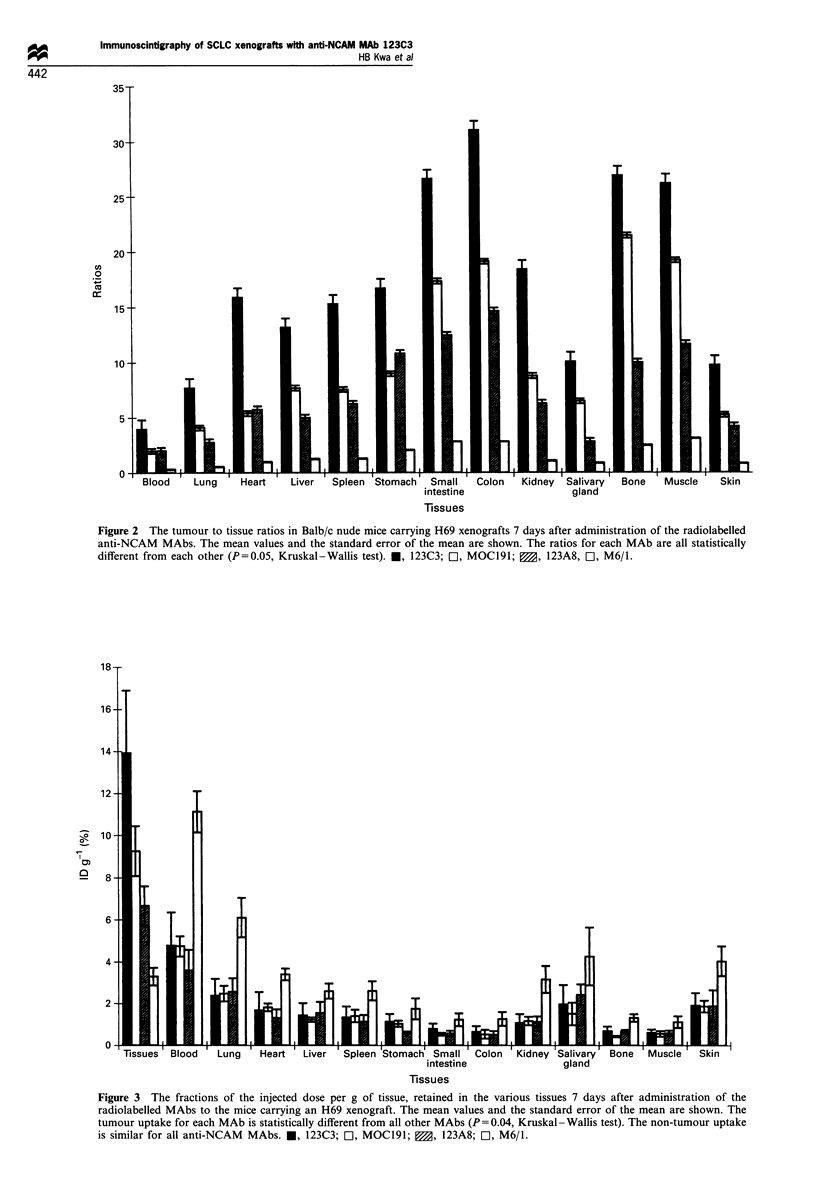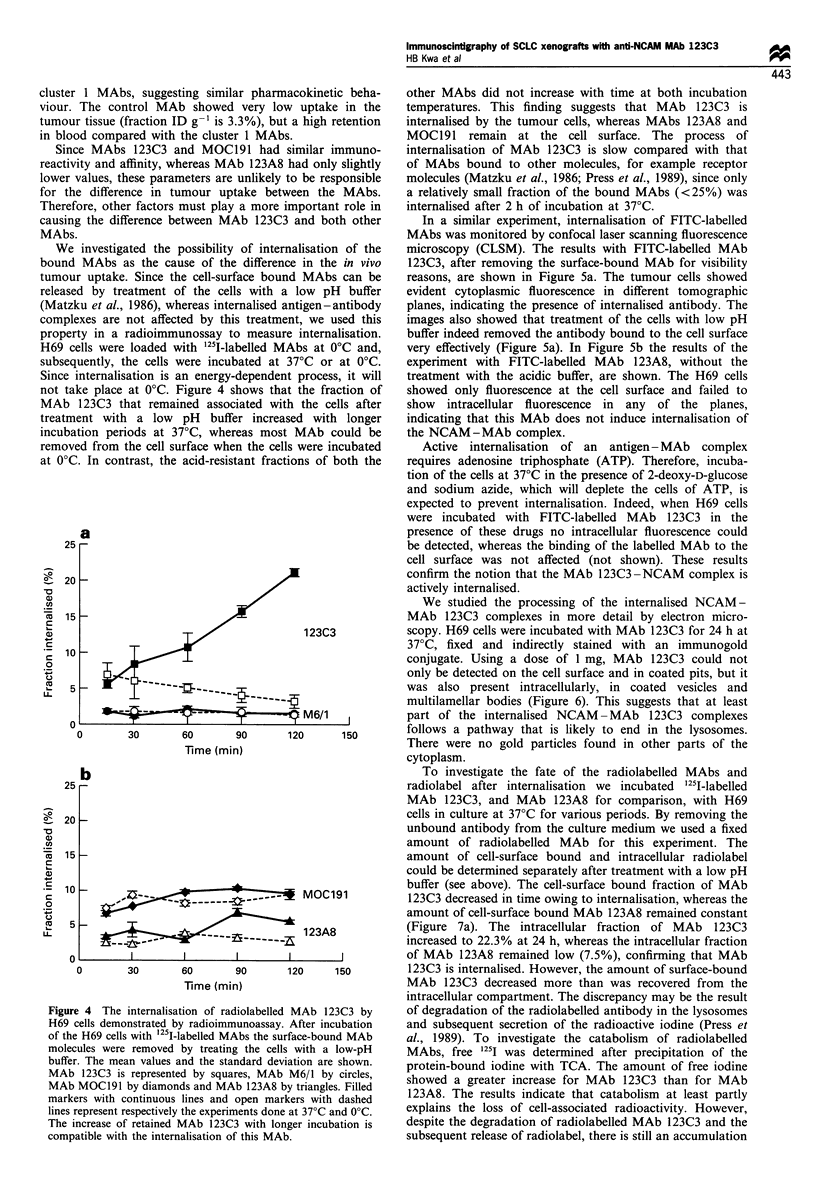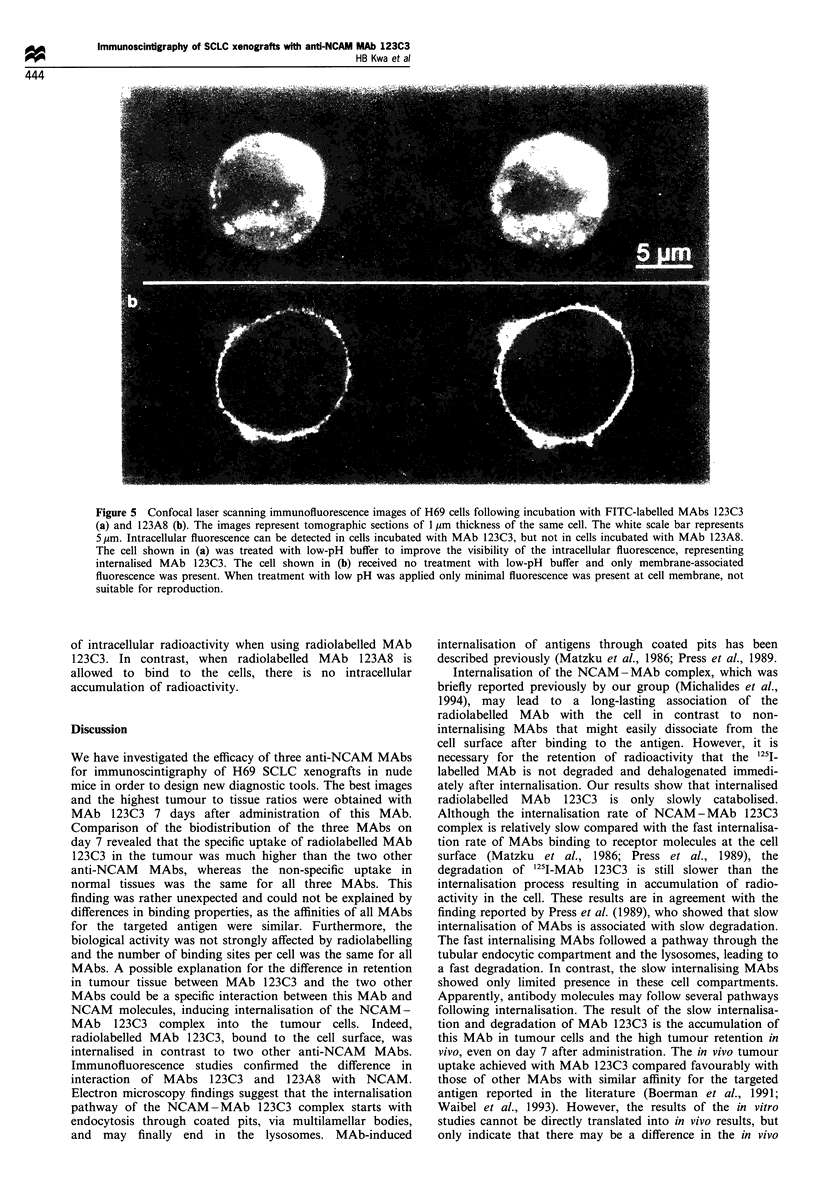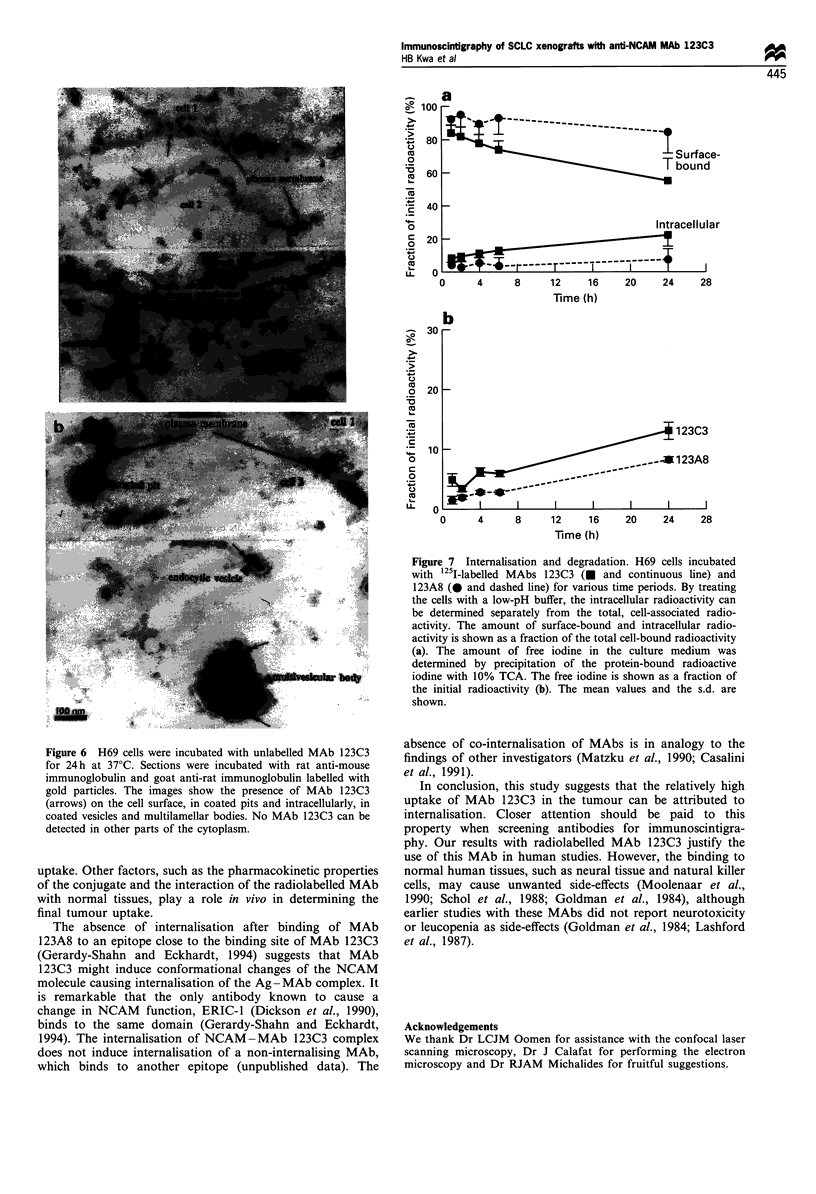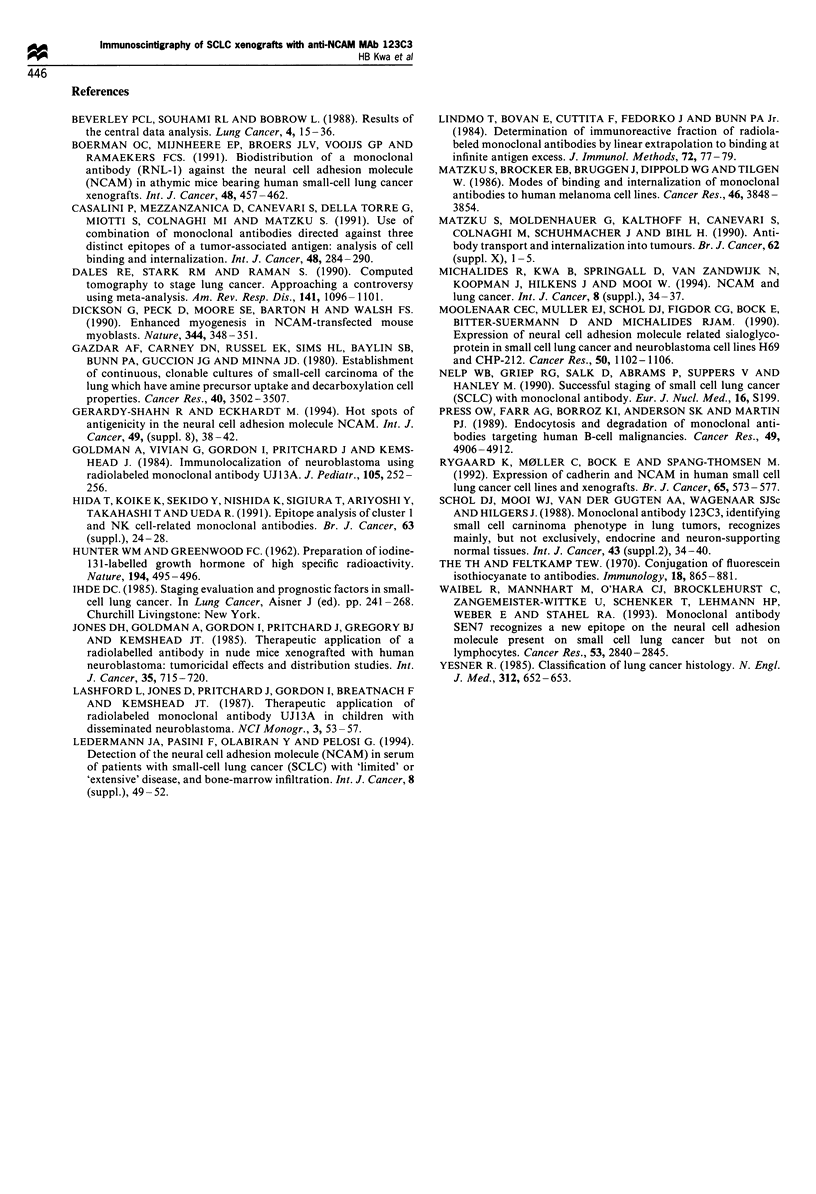# Immunoscintigraphy of small-cell lung cancer xenografts with anti neural cell adhesion molecule monoclonal antibody, 123C3: improvement of tumour uptake by internalisation.

**DOI:** 10.1038/bjc.1996.79

**Published:** 1996-02

**Authors:** H. B. Kwa, J. Wesseling, A. H. Verhoeven, N. van Zandwijk, J. Hilkens

**Affiliations:** Department of Tumour Biology, The Netherlands Cancer Institute, Amsterdam, The Netherlands.

## Abstract

**Images:**


British Journal of Cancer (1996) 73, 439-446
© 1996 Stockton Press All rights reserved 0007-0920/96 $12.00

### Immunoscintigraphy of small-cell lung cancer xenografts with anti neural cell adhesion molecule monoclonal antibody, 123C3: improvement of tumour uptake by internalisation

HB Kwa[1,2], J Wesseling[1], AHM Verhoeven[1], N van Zandwijk[2] and J Hilkens[1]

*Departments of [1]Tumour Biology and [2]Medical Oncology, The Netherlands Cancer Institute (Antoni van Leeuwenhoekhuis), Plesmanlaan 121, 1066 CX Amsterdam, The Netherlands.*

**Summary** The efficacy of three murine monoclonal antibodies (MAbs) for immunoscintigraphy of small-cell lung cancer (SCLC) xenografts was studied in a Balb/c *nu/nu* mouse model. These MAbs, 123C3, 123A8 and MOC191, belong to cluster 1 of anti-SCLC MAbs and bind to the neural cell adhesion molecule (NCAM) with similar affinity. After intraperitoneal injection of these MAbs, labelled with $^{125}I$, the highest uptake in tumour tissue was obtained with MAb 123C3. Seven days after administration of this MAb 13.9% of the injected dose per gram of tumour tissue was retained in the tumour. The corresponding tumour to tissue ratios ranged from 3.97 for blood to 31.03 for colon. The imaging results and the tumour uptake were less favourable for the two other MAbs, 123A8 and MOC191 (fractions of injected dose respectively 6.7% and 9.2%), although affinity, biological activity after labelling and uptake in non-tumour tissues were very similar for all three MAbs. These results may be explained by the differences in the interaction between the MAbs and the tumour cells. MAb 123C3 is internalised into tumour cells, whereas both other anti-NCAM MAbs are not. Internalisation into NCI H69 cells was demonstrated *in vitro* by a radioimmunoassay, confocal laser scanning microscopy and electron microscopy. The internalised fraction of MAb 123C3 was 22.3% after 24 h, whereas this fraction was only 7.5% for MAb 123A8. Although the internalised radiolabelled MAbs are usually degraded and dehalogenated intracellularly, the retained radioactivity is high. Apparently, intracellular degradation of radiolabelled MAb 123C3 and subsequent secretion of radioactive iodine did not prevent the accumulation of intracellular radioactivity. In conclusion, accumulation and retention of radioactivity in the tumour tissue, due to internalisation of radiolabelled MAbs, may improve the results of immunoscintigraphy.

**Keywords:** immunoscintigraphy; small-cell lung cancer; neural cell adhesion molecule; monoclonal antibody; internalisation

Clinically, lung cancer is divided into small-cell lung cancer (SCLC) and non-small-cell lung cancer (NSCLC). SCLC accounts for about 25% of the cases and is associated with the worst prognosis of all lung cancers (Yesner, 1985). For the prognosis and treatment of this type of tumour it is very important to determine the initial stage of the tumour (Ihde, 1985). Unfortunately, standard staging procedure is very laborious and inaccurate. In a recent meta-analysis, the best non-invasive diagnostic technique, computerised tomography (CT), achieved an accuracy of only 0.80 for the detection of intrathoracic lesions (Dales *et al.*, 1990). There is clearly a need for new techniques with a higher sensitivity and specificity to replace the multitude of diagnostic procedures used in the initial staging (Ihde, 1985). Monoclonal antibodies (MAbs) have greatly increased the sensitivity in detecting bone marrow metastases in SCLC (Ledermann *et al.*, 1994). Using radiolabelled MAbs in patients with SCLC (Nelp *et al.*, 1990) distant metastases can be detected by immunoscintigraphy in 10% of patients with limited disease, when staged by conventional methods. This finding suggests that immunoscintigraphy has a higher sensitivity than the standard staging procedure.

Monoclonal antibodies (MAbs) raised against SCLC are categorised in clusters by the antigen recognised, according to an international workshop (Beverley *et al.*, 1988). The MAbs belonging to cluster 1 bind to the neural cell adhesion molecule (NCAM) (Moolenaar *et al.*, 1990). Of the existing NCAM isoforms, NCAM-140 and NCAM-180 are the most important in SCLC (Moolenaar *et al.*, 1990) and all three cluster 1 MAbs used in this study recognise these isoforms (Beverley *et al.*, 1988; Hida *et al.*, 1991). As NCAM is expressed by all SCLCs, it seems a suitable target for immunoscintigraphy with these MAbs. The three MAbs investigated in this study showed similar affinities for NCAM and were used for radioimmunodetection in a mouse SCLC xenograft model. We demonstrated that one of the MAbs that is internalised by the SCLC cells shows a significantly higher uptake in tumour tissue.

Correspondence: HB Kwa, Department of Pulmonology, OLVG Hospital, 1e Oosterparkstraat 279, 1091 HM Amsterdam, The Netherlands
Received 16 May 1995; revised 8 September 1995; accepted 13 September 1995

### Materials and methods

### *Cell lines*

The NCI H69 cell line (hereafter referred to as H69 cells), derived from a small cell lung carcinoma, was kindly provided by Dr D Carney (Gazdar *et al.*, 1980) and was grown in Dulbecco's modified Eagle medium supplemented with 1 mM glutamine, 10% fetal calf serum and antibiotics. This cell line expresses high levels of NCAM when grown *in vitro* and as xenografts in nude mice (Rygaard *et al.*, 1992).

### *Monoclonal antibodies*

From a panel of nine cluster 1 MAbs, MAbs 123C3, 123A8 and MOC191 were selected for this study, on the basis of their affinity for NCAM and biological activity after labelling with radioactive iodine (Beverley *et al.*, 1988; Moolenaar *et al.*, 1990). MAbs 123C3 and 123A8 are IgG1 antibodies raised at our institute against a membrane fraction of a fresh SCLC specimen. Both MAbs recognise epitopes on the protein backbone of NCAM close to the attachment site of the polysialic acid side-chains (Gerardy-Shahn and Eckhardt, 1994) and bind to all NCAM isoforms (Moolenaar *et al.*, 1990). The tissue distribution of MAb 123C3 in human tissues was described previously (Schol *et al.*, 1988). MAb 123A8 showed a very similar tissue distribution (DJ Schol and Ph C Hageman, unpublished data). MAb MOC191 is an

IgG2a antibody and was kindly provided by Dr LF de Ley, University Hospital of Groningen. The epitope recognised by MAb MOC191 is probably located at the third immunoglobulin loop (Hida *et al.*, 1991; Gerardy-Shahn and Eckhardt, 1994). MAb M6/1, and IgG1 MAb raised against melanoma cells, detects a high molecular weight proteoglycan and did not bind to H69 cells *in vitro*. All MAbs were affinity purified from ascitic fluid by protein-A-Sephadex column chromatography and were eluted with a citrate buffer (pH 4.5) (Jones *et al.*, 1985).

*Antibody labelling*

The MAbs were labelled with $^{125}$I (Amersham) using the chloramine-T method (Hunter and Greenwood, 1962). MAb (50 $\mu$g) was labelled with 50 $\mu$Ci of $^{125}$I. Free iodine was removed from the labelled MAbs by ion-exchange column chromatography (Dowex G25). For immunofluorescence experiments the MAbs were labelled with fluorescein isothiocyanate (FITC) (The and Feltkamp, 1970). Briefly, 1 mg of FITC in dimethylsulphoxide (DMSO) was added to 1 mg of MAb solution in 0.1 M sodium carbonate buffer (pH 9.5) and incubated overnight at 4°C. Free FITC was removed over a Dowex G25 column.

*Determination of immunoreactivity and* in vitro *affinity*

The immunoreactivity of the MAbs after labelling with $^{125}$I, was assessed according to the method described by Lindmo *et al.* (1984) with slight modifications. Serial dilutions of H69 cells in a volume of 200 $\mu$l of medium, starting at a cell concentration of $25 \times 10^6$ ml$^{-1}$, were incubated with an equivolume of $^{125}$I-labelled MAb in phosphate-buffered saline (PBS) at a concentration of 50 ng ml$^{-1}$ for 4 h at 4°C. After centrifugation of the cell suspension 200 $\mu$l of supernatant was taken and the fraction with and without the pellet was measured separately in a gamma counter to determine the amount of bound and free radiolabelled antibody. From the results the immunoreactive fraction after iodination was calculated and the immunoreactive fraction was used to determine the affinity using the Scatchard method (Lindmo *et al.*, 1984). Briefly, 200 $\mu$l of a cell suspension in medium containing $2.5 \times 10^6$ H69 cells was incubated for 4 h at 4°C with 200 $\mu$l of a serial dilution of the radiolabelled MAb in PBS starting at a concentration of 1 $\mu$g ml$^{-1}$. The amount of bound and free radioactivity was determined in the same way as described above. After correction for the immunoreactive fraction the association constant $K_a$ and the number of binding sites per cell were calculated.

*H69 xenograft model*

Human xenografts of H69 cells were established in Balb/c-*nu*/*nu* mice by subcutaneous injection of a cell suspension in PBS containing $10^6$ cells from *in vitro* cultures. Within 3 to 4 weeks the tumours reached a volume of 150–500 mm$^3$ (diameters between 5 mm and 10 mm), suitable for *in vivo* studies.

*Immunoscintigraphy and biolocalisation*

For imaging purposes, groups of five tumour-bearing mice were injected with radiolabelled MAbs 123C3, 123A8 or MOC191 and a group of three mice received an injection of radiolabelled MAb M6/1. A dose varying between 50 and 100 $\mu$g of MAb labelled with 50 $\mu$Ci $^{125}$I was injected intraperitoneally in each mouse. No potassium iodide was given to the animals to block the iodine uptake in the thyroid gland. Images were made on days 2, 4 and 7 after administration of the radiolabelled MAb. From the images the counts from the tumour area and the background are used for quantification of the tumour to background ratio. The animals were killed after the last image had been made, and the tissue samples were collected. Wet tissue weight was determined and the retained radioactivity in the samples was measured in a gamma counter. Percentages of injected dose per g of tissue and tumour to tissue ratios for the samples were calculated from these results.

*Internalisation assay*

A cell suspension containing $1 \times 10^6$ H69 cells in 200 $\mu$l of medium was incubated with 10–20 $\mu$g of each MAb in 200 $\mu$l of PBS, labelled with 50 $\mu$Ci $^{125}$I, at 4°C for 60 min. After removing unbound antibodies by washing the cells three times in PBS the bound radioactivity was determined in a gamma counter. Subsequently, the cells were incubated at 0°C or 37°C for various time periods and washed three times in PBS. The antibodies still present on the cell surface were removed by incubation of the cells with a buffer containing 0.1 M glycine hydrochloric acid and 0.1 M acetic acid (pH 3) for 5 min at 0°C (modified from Matzku *et al.*, 1986). After washing the cells three times in PBS, the remaining radioactivity was measured. The fraction of internalised antibody was calculated from the remaining radioactivity divided by the initially bound radioactivity.

*Immunofluorescence*

To determine the internalisation of MAb 123C3 by immunofluorescence, $1 \times 10^6$ H69 cells in 1 ml of medium were incubated with 20 $\mu$g FITC-labelled MAb 123C3 for 120 min at 37°C. Subsequently, the cells were washed with PBS and divided into two fractions. One fraction did not receive any additional treatment and from the other fraction the surface-bound antibody was removed by treatment with low-pH buffer as described above to improve visibility of the intracellular fraction. Then, the cells were attached to a glass slide coated with poly-L-lysin and fixed with 4% paraformaldehyde for 10 min. As control, the same experiment was done with FITC-labelled MAb 123A8 and with MAb 123C3 in the presence of 50 mM 2-deoxy-D-glucose and 0.01% sodium azide to block energy-dependent processes, including internalisation. The cells were imaged with a confocal laser scanning fluorescence microscope (CLSM) with tomographic slices of 1 $\mu$m thickness.

*Electron microscopy*

To investigate the internalisation process at the ultrastructural level, a suspension of $1 \times 10^8$ H69 cells in 1 ml was incubated with 1 mg of MAb 123C3 for 24 h. Unbound MAb was removed by washing the cells three times in PBS. The cells were fixed with 4% paraformaldehyde in 0.1 M phosphate buffer for 60 min at room temperature and embedded in 10% gelatine. After impregnation with 20% PVP-10 and 1.8 M sucrose for 120 min, the cells were frozen in liquid nitrogen and 60 nm sections were cut. Subsequently, the sections were incubated with a 1:40 dilution of rat- anti-mouse immunoglobulin [RAM/Ig (Nordic)] for 30 min, followed by incubation a 1:40 dilution of a conjugate of goat anti-rat immunoglobulin with gold particles [GAR/G10 (Amersham)] for 20 min. After washing, the sections were covered with 1.5% methylcellulose and 0.3% uranylacetate. The sections were then examined with a Philips CM10 electron microscope.

*Internalisation and degradation*

To investigate the fate of the radiolabelled antibody after internalisation, 2.5 $\mu$g MAb 123C3 or 123A8 in PBS, labelled with 25 $\mu$Ci $^{125}$I, were incubated with $1.4 \times 10^6$ H69 cells in 0.5 ml of culture medium at 4°C for 1 h. After removal of the unbound MAbs, the total amount of radioactivity in the cell suspension was determined and the cells were cultured at 37°C. After several periods the culture medium was collected and the cell suspensions were washed twice in PBS. The amount of radioactivity in the culture medium and the cell-associated radioactivity were determined in a gamma counter.

Cell-surface bound and intracellular radioactivity were determined by measuring the cell-associated radioactivity before and after treatment with low-pH buffer (see above). The amount of free $^{125}I$ in the culture supernatant was dertermined by adding 0.3 ml of 10% trichloroacetic acid (TCA) and counting the radioactivity in the supernatant after centrifugation.

### Results

We determined the immunoreactive fraction of a panel of nine cluster 1 MAbs after labelling with $^{125}I$ according to the method described by Lindmo *et al.* (1984). The affinity of the MAbs for NCAM was determined by Scatchard assay. For these assays, H69 SCLC cells, expressing high levels of NCAM, were used (Rygaard *et al.*, 1992). MAbs 123C3, 123A8 and MOC191, which showed the highest immunoreactivity and affinity, were selected for further study. The immunoreactivity of these MAbs were respectively 0.94, 0.68 and 0.85 and the association constants ($K_a$), corrected for the immunoreactive fraction, were respectively 1.04, 0.43 and $1.16.10^9$ $M^{-1}$. The results of 3–5 experiments were shown. From the results of the Scatchard analysis we calculated that for each MAb approximately the same numbers of antibody binding sites were available per H69 cell ($5 \times 10^6$ per cell).

All three selected MAbs and the control MAb, M6/1, labelled with $^{125}I$, were injected intraperitoneally into mice bearing H69 xenografts and after 2, 4 and 7 days scintigrams were made. The images made on days 2 and 4 showed a high background and a relatively low tumour uptake, whereas the images made on day 7 had a relatively low background and higher tumour uptake and were judged to be optimal (Figure 1). These results were confirmed by quantifying the counts from the images. The mean tumour to background ratio obtained from the images made with MAb 123C3 showed an increase from 1.03 on day 2 to 1.99 on day 7, whereas for MAbs MOC191 and 123A8 the values remained constant at 1.13 and 1.05. Images made with radiolabelled MAb 123C3 showed a higher tumour uptake, resulting in a more distinct localisation of the tumour than the images produced with MAbs MOC191 and 123A8. The larger tumours showed a higher total radioactive count than the smaller ones. Images made with M6/1, the control antibody, showed no tumour at all, and a higher background activity than the cluster 1 MAbs. The thyroid gland was not blocked in order to facilitate the orientation of the scan and was clearly visible on all scintigraphic images.

On day 7 the mice were dissected and the radioactivity in each tissue was measured. The total radioactive counts varied greatly with tumour size, but all calculations for tumour tissue ratios and fraction of injected dose were done on the counts per g of tissue, which showed less variation. The mean tumour to tissue ratios 7 days after administration of the radiolabelled MAbs are shown in Figure 2. The highest tumour to tissue ratios were observed with MAb 123C3. The tumour to blood ratio achieved with this MAb was the highest (3.97, $P=0.05$, Kruskal–Wallis test), twice the ratio for MAbs MOC191 and 123A8 (respectively 1.99 and 2.00). The control MAb, M6/1, showed very low ratios for all tissues tested (tumour to blood ratio 0.29). The mean fractions of the injected dose retained in the tissues on day 7 after administration of the MAbs are shown in Figure 3. MAb 123C3 revealed the highest uptake in the tumour tissue (13.9%, $P=0.04$, Kruskal–Wallis test), whereas the values for MAbs MOC191 and 123A8 were significantly lower (9.2% and 6.7%). The fraction of the injected dose in the non-tumour tissues was very similar for all three tested

**Figure 1** Immunoscintigrams of H69 xenografts in Balb/c *nu*/*nu* mice using three anti-NCAM MAbs and the control antibody M6/1. The images were made 7 days after intraperitoneal administration of $^{125}I$-labelled MAbs 123C3 (**a**), MOC191 (**b**), 123A8 (**c**) and MAb M6/1 (**d**). The images of three mice are shown except in (**b**), on which only two mice are shown. On the scans the heads of the mice are directed upwards. The xenografts are indicated by arrows in (**a**–**c**) and are located in the side of the mice. In (**d**) the arrow indicates the location of the thyroid, which is not blocked to facilitate the orientation of the images.

**Figure 2** The tumour to tissue ratios in Balb/c nude mice carrying H69 xenografts 7 days after administration of the radiolabelled anti-NCAM MAbs. The mean values and the standard error of the mean are shown. The ratios for each MAb are all statistically different from each other ($P=0.05$, Kruskal–Wallis test). ■, 123C3; □, MOC191; ▨, 123A8, □, M6/1.

**Figure 3** The fractions of the injected dose per g of tissue, retained in the various tissues 7 days after administration of the radiolabelled MAbs to the mice carrying an H69 xenograft. The mean values and the standard error of the mean are shown. The tumour uptake for each MAb is statistically different from all other MAbs ($P=0.04$, Kruskal–Wallis test). The non-tumour uptake is similar for all anti-NCAM MAbs. ■, 123C3; □, MOC191; ▨, 123A8; □, M6/1.

cluster 1 MAbs, suggesting similar pharmacokinetic behaviour. The control MAb showed very low uptake in the tumour tissue (fraction ID $g^{-1}$ is 3.3%), but a high retention in blood compared with the cluster 1 MAbs.

Since MAbs 123C3 and MOC191 had similar immunoreactivity and affinity, whereas MAb 123A8 had only slightly lower values, these parameters are unlikely to be responsible for the difference in tumour uptake between the MAbs. Therefore, other factors must play a more important role in causing the difference between MAb 123C3 and both other MAbs.

We investigated the possibility of internalisation of the bound MAbs as the cause of the difference in the *in vivo* tumour uptake. Since the cell-surface bound MAbs can be released by treatment of the cells with a low pH buffer (Matzku *et al.*, 1986), whereas internalised antigen–antibody complexes are not affected by this treatment, we used this property in a radioimmunossay to measure internalisation. H69 cells were loaded with $^{125}$I-labelled MAbs at 0°C and, subsequently, the cells were incubated at 37°C or at 0°C. Since internalisation is an energy-dependent process, it will not take place at 0°C. Figure 4 shows that the fraction of MAb 123C3 that remained associated with the cells after treatment with a low pH buffer increased with longer incubation periods at 37°C, whereas most MAb could be removed from the cell surface when the cells were incubated at 0°C. In contrast, the acid-resistant fractions of both the other MAbs did not increase with time at both incubation temperatures. This finding suggests that MAb 123C3 is internalised by the tumour cells, whereas MAbs 123A8 and MOC191 remain at the cell surface. The process of internalisation of MAb 123C3 is slow compared with that of MAbs bound to other molecules, for example receptor molecules (Matzku *et al.*, 1986; Press *et al.*, 1989), since only a relatively small fraction of the bound MAbs (<25%) was internalised after 2 h of incubation at 37°C.

**Figure 4** The internalisation of radiolabelled MAb 123C3 by H69 cells demonstrated by radioimmunoassay. After incubation of the H69 cells with $^{125}$I-labelled MAbs the surface-bound MAb molecules were removed by treating the cells with a low-pH buffer. The mean values and the standard deviation are shown. MAb 123C3 is represented by squares, MAb M6/1 by circles, MAb MOC191 by diamonds and MAb 123A8 by triangles. Filled markers with continuous lines and open markers with dashed lines represent respectively the experiments done at 37°C and 0°C. The increase of retained MAb 123C3 with longer incubation is compatible with the internalisation of this MAb.

In a similar experiment, internalisation of FITC-labelled MAbs was monitored by confocal laser scanning fluorescence microscopy (CLSM). The results with FITC-labelled MAb 123C3, after removing the surface-bound MAb for visibility reasons, are shown in Figure 5a. The tumour cells showed evident cytoplasmic fluorescence in different tomographic planes, indicating the presence of internalised antibody. The images also showed that treatment of the cells with low pH buffer indeed removed the antibody bound to the cell surface very effectively (Figure 5a). In Figure 5b the results of the experiment with FITC-labelled MAb 123A8, without the treatment with the acidic buffer, are shown. The H69 cells showed only fluorescence at the cell surface and failed to show intracellular fluorescence in any of the planes, indicating that this MAb does not induce internalisation of the NCAM–MAb complex.

Active internalisation of an antigen–MAb complex requires adenosine triphosphate (ATP). Therefore, incubation of the cells at 37°C in the presence of 2-deoxy-D-glucose and sodium azide, which will deplete the cells of ATP, is expected to prevent internalisation. Indeed, when H69 cells were incubated with FITC-labelled MAb 123C3 in the presence of these drugs no intracellular fluorescence could be detected, whereas the binding of the labelled MAb to the cell surface was not affected (not shown). These results confirm the notion that the MAb 123C3–NCAM complex is actively internalised.

We studied the processing of the internalised NCAM–MAb 123C3 complexes in more detail by electron microscopy. H69 cells were incubated with MAb 123C3 for 24 h at 37°C, fixed and indirectly stained with an immunogold conjugate. Using a dose of 1 mg, MAb 123C3 could not only be detected on the cell surface and in coated pits, but it was also present intracellularly, in coated vesicles and multilamellar bodies (Figure 6). This suggests that at least part of the internalised NCAM–MAb 123C3 complexes follows a pathway that is likely to end in the lysosomes. There were no gold particles found in other parts of the cytoplasm.

To investigate the fate of the radiolabelled MAbs and radiolabel after internalisation we incubated $^{125}$I-labelled MAb 123C3, and MAb 123A8 for comparison, with H69 cells in culture at 37°C for various periods. By removing the unbound antibody from the culture medium we used a fixed amount of radiolabelled MAb for this experiment. The amount of cell-surface bound and intracellular radiolabel could be determined separately after treatment with a low pH buffer (see above). The cell-surface bound fraction of MAb 123C3 decreased in time owing to internalisation, whereas the amount of cell-surface bound MAb 123A8 remained constant (Figure 7a). The intracellular fraction of MAb 123C3 increased to 22.3% at 24 h, whereas the intracellular fraction of MAb 123A8 remained low (7.5%), confirming that MAb 123C3 is internalised. However, the amount of surface-bound MAb 123C3 decreased more than was recovered from the intracellular compartment. The discrepancy may be the result of degradation of the radiolabelled antibody in the lysosomes and subsequent secretion of the radioactive iodine (Press *et al.*, 1989). To investigate the catabolism of radiolabelled MAbs, free $^{125}$I was determined after precipitation of the protein-bound iodine with TCA. The amount of free iodine showed a greater increase for MAb 123C3 than for MAb 123A8. The results indicate that catabolism at least partly explains the loss of cell-associated radioactivity. However, despite the degradation of radiolabelled MAb 123C3 and the subsequent release of radiolabel, there is still an accumulation

**Figure 5** Confocal laser scanning immunofluorescence images of H69 cells following incubation with FITC-labelled MAbs 123C3 (**a**) and 123A8 (**b**). The images represent tomographic sections of 1 μm thickness of the same cell. The white scale bar represents 5 μm. Intracellular fluorescence can be detected in cells incubated with MAb 123C3, but not in cells incubated with MAb 123A8. The cell shown in (**a**) was treated with low-pH buffer to improve the visibility of the intracellular fluorescence, representing internalised MAb 123C3. The cell shown in (**b**) received no treatment with low-pH buffer and only membrane-associated fluorescence was present. When treatment with low pH was applied only minimal fluorescence was present at cell membrane, not suitable for reproduction.

of intracellular radioactivity when using radiolabelled MAb 123C3. In contrast, when radiolabelled MAb 123A8 is allowed to bind to the cells, there is no intracellular accumulation of radioactivity.

### Discussion

We have investigated the efficacy of three anti-NCAM MAbs for immunoscintigraphy of H69 SCLC xenografts in nude mice in order to design new diagnostic tools. The best images and the highest tumour to tissue ratios were obtained with MAb 123C3 7 days after administration of this MAb. Comparison of the biodistribution of the three MAbs on day 7 revealed that the specific uptake of radiolabelled MAb 123C3 in the tumour was much higher than the two other anti-NCAM MAbs, whereas the non-specific uptake in normal tissues was the same for all three MAbs. This finding was rather unexpected and could not be explained by differences in binding properties, as the affinities of all MAbs for the targeted antigen were similar. Furthermore, the biological activity was not strongly affected by radiolabelling and the number of binding sites per cell was the same for all MAbs. A possible explanation for the difference in retention in tumour tissue between MAb 123C3 and the two other MAbs could be a specific interaction between this MAb and NCAM molecules, inducing internalisation of the NCAM–MAb 123C3 complex into the tumour cells. Indeed, radiolabelled MAb 123C3, bound to the cell surface, was internalised in contrast to two other anti-NCAM MAbs. Immunofluorescence studies confirmed the difference in interaction of MAbs 123C3 and 123A8 with NCAM. Electron microscopy findings suggest that the internalisation pathway of the NCAM–MAb 123C3 complex starts with endocytosis through coated pits, via multilamellar bodies, and may finally end in the lysosomes. MAb-induced internalisation of antigens through coated pits has been described previously (Matzku *et al.*, 1986; Press *et al.*, 1989.

Internalisation of the NCAM–MAb complex, which was briefly reported previously by our group (Michalides *et al.*, 1994), may lead to a long-lasting association of the radiolabelled MAb with the cell in contrast to non-internalising MAbs that might easily dissociate from the cell surface after binding to the antigen. However, it is necessary for the retention of radioactivity that the $^{125}$I-labelled MAb is not degraded and dehalogenated immediately after internalisation. Our results show that internalised radiolabelled MAb 123C3 is only slowly catabolised. Although the internalisation rate of NCAM–MAb 123C3 complex is relatively slow compared with the fast internalisation rate of MAbs binding to receptor molecules at the cell surface (Matzku *et al.*, 1986; Press *et al.*, 1989), the degradation of $^{125}$I-MAb 123C3 is still slower than the internalisation process resulting in accumulation of radioactivity in the cell. These results are in agreement with the finding reported by Press *et al.* (1989), who showed that slow internalisation of MAbs is associated with slow degradation. The fast internalising MAbs followed a pathway through the tubular endocytic compartment and the lysosomes, leading to a fast degradation. In contrast, the slow internalising MAbs showed only limited presence in these cell compartments. Apparently, antibody molecules may follow several pathways following internalisation. The result of the slow internalisation and degradation of MAb 123C3 is the accumulation of this MAb in tumour cells and the high tumour retention *in vivo*, even on day 7 after administration. The *in vivo* tumour uptake achieved with MAb 123C3 compared favourably with those of other MAbs with similar affinity for the targeted antigen reported in the literature (Boerman *et al.*, 1991; Waibel *et al.*, 1993). However, the results of the *in vitro* studies cannot be directly translated into *in vivo* results, but only indicate that there may be a difference in the *in vivo*

**Figure 6** H69 cells were incubated with unlabelled MAb 123C3 for 24 h at 37°C. Sections were incubated with rat anti-mouse immunoglobulin and goat anti-rat immunoglobulin labelled with gold particles. The images show the presence of MAb 123C3 (arrows) on the cell surface, in coated pits and intracellularly, in coated vesicles and multilamellar bodies. No MAb 123C3 can be detected in other parts of the cytoplasm.

**Figure 7** Internalisation and degradation. H69 cells incubated with $^{125}$I-labelled MAbs 123C3 (■ and continuous line) and 123A8 (● and dashed line) for various time periods. By treating the cells with a low-pH buffer, the intracellular radioactivity can be determined separately from the total, cell-associated radioactivity. The amount of surface-bound and intracellular radioactivity is shown as a fraction of the total cell-bound radioactivity (**a**). The amount of free iodine in the culture medium was determined by precipitation of the protein-bound radioactive iodine with 10% TCA. The free iodine is shown as a fraction of the initial radioactivity (**b**). The mean values and the s.d. are shown.

uptake. Other factors, such as the pharmacokinetic properties of the conjugate and the interaction of the radiolabelled MAb with normal tissues, play a role *in vivo* in determining the final tumour uptake.

The absence of internalisation after binding of MAb 123A8 to an epitope close to the binding site of MAb 123C3 (Gerardy-Shahn and Eckhardt, 1994) suggests that MAb 123C3 might induce conformational changes of the NCAM molecule causing internalisation of the Ag–MAb complex. It is remarkable that the only antibody known to cause a change in NCAM function, ERIC-1 (Dickson *et al*., 1990), binds to the same domain (Gerardy-Shahn and Eckhardt, 1994). The internalisation of NCAM–MAb 123C3 complex does not induce internalisation of a non-internalising MAb, which binds to another epitope (unpublished data). The absence of co-internalisation of MAbs is in analogy to the findings of other investigators (Matzku *et al*., 1990; Casalini *et al*., 1991).

In conclusion, this study suggests that the relatively high uptake of MAb 123C3 in the tumour can be attributed to internalisation. Closer attention should be paid to this property when screening antibodies for immunoscintigraphy. Our results with radiolabelled MAb 123C3 justify the use of this MAb in human studies. However, the binding to normal human tissues, such as neural tissue and natural killer cells, may cause unwanted side-effects (Moolenaar *et al*., 1990; Schol *et al*., 1988; Goldman *et al*., 1984), although earlier studies with these MAbs did not report neurotoxicity or leucopenia as side-effects (Goldman *et al*., 1984; Lashford *et al*., 1987).

**Acknowledgements**
We thank Dr LCJM Oomen for assistance with the confocal laser scanning microscopy, Dr J Calafat for performing the electron microscopy and Dr RJAM Michalides for fruitful suggestions.

### References

BEVERLEY PCL, SOUHAMI RL AND BOBROW L. (1988). Results of the central data analysis. *Lung Cancer*, **4**, 15–36.

BOERMAN OC, MIJNHEERE EP, BROERS JLV, VOOIJS GP AND RAMAEKERS FCS. (1991). Biodistribution of a monoclonal antibody (RNL-1) against the neural cell adhesion molecule (NCAM) in athymic mice bearing human small-cell lung cancer xenografts. *Int. J. Cancer*, **48**, 457–462.

CASALINI P, MEZZANZANICA D, CANEVARI S, DELLA TORRE G, MIOTTI S, COLNAGHI MI AND MATZKU S. (1991). Use of combination of monoclonal antibodies directed against three distinct epitopes of a tumor-associated antigen: analysis of cell binding and internalization. *Int. J. Cancer*, **48**, 284–290.

DALES RE, STARK RM AND RAMAN S. (1990). Computed tomography to stage lung cancer. Approaching a controversy using meta-analysis. *Am. Rev. Resp. Dis.*, **141**, 1096–1101.

DICKSON G, PECK D, MOORE SE, BARTON H AND WALSH FS. (1990). Enhanced myogenesis in NCAM-transfected mouse myoblasts. *Nature*, **344**, 348–351.

GAZDAR AF, CARNEY DN, RUSSEL EK, SIMS HL, BAYLIN SB, BUNN PA, GUCCION JG AND MINNA JD. (1980). Establishment of continuous, clonable cultures of small-cell carcinoma of the lung which have amine precursor uptake and decarboxylation cell properties. *Cancer Res.*, **40**, 3502–3507.

GERARDY-SHAHN R AND ECKHARDT M. (1994). Hot spots of antigenicity in the neural cell adhesion molecule NCAM. *Int. J. Cancer*, **49**, (suppl. 8), 38–42.

GOLDMAN A, VIVIAN G, GORDON I, PRITCHARD J AND KEMSHEAD J. (1984). Immunolocalization of neuroblastoma using radiolabeled monoclonal antibody UJ13A. *J. Pediatr.*, **105**, 252–256.

HIDA T, KOIKE K, SEKIDO Y, NISHIDA K, SIGIURA T, ARIYOSHI Y, TAKAHASHI T AND UEDA R. (1991). Epitope analysis of cluster 1 and NK cell-related monoclonal antibodies. *Br. J. Cancer*, **63** (suppl.), 24–28.

HUNTER WM AND GREENWOOD FC. (1962). Preparation of iodine-131-labelled growth hormone of high specific radioactivity. *Nature*, **194**, 495–496.

IHDE DC. (1985). Staging evaluation and prognostic factors in small-cell lung cancer. In *Lung Cancer*, Aisner J (ed). pp. 241–268. Churchill Livingstone: New York.

JONES DH, GOLDMAN A, GORDON I, PRITCHARD J, GREGORY BJ AND KEMSHEAD JT. (1985). Therapeutic application of a radiolabelled antibody in nude mice xenografted with human neuroblastoma: tumoricidal effects and distribution studies. *Int. J. Cancer*, **35**, 715–720.

LASHFORD L, JONES D, PRITCHARD J, GORDON I, BREATNACH F AND KEMSHEAD JT. (1987). Therapeutic application of radiolabeled monoclonal antibody UJ13A in children with disseminated neuroblastoma. *NCI Monogr.*, **3**, 53–57.

LEDERMANN JA, PASINI F, OLABIRAN Y AND PELOSI G. (1994). Detection of the neural cell adhesion molecule (NCAM) in serum of patients with small-cell lung cancer (SCLC) with 'limited' or 'extensive' disease, and bone-marrow infiltration. *Int. J. Cancer*, **8** (suppl.), 49–52.

LINDMO T, BOVAN E, CUTTITA F, FEDORKO J AND BUNN PA Jr. (1984). Determination of immunoreactive fraction of radiolabeled monoclonal antibodies by linear extrapolation to binding at infinite antigen excess. *J. Immunol. Methods*, **72**, 77–79.

MATZKU S, BROCKER EB, BRUGGEN J, DIPPOLD WG AND TILGEN W. (1986). Modes of binding and internalization of monoclonal antibodies to human melanoma cell lines. *Cancer Res.*, **46**, 3848–3854.

MATZKU S, MOLDENHAUER G, KALTHOFF H, CANEVARI S, COLNAGHI M, SCHUHMACHER J AND BIHL H. (1990). Antibody transport and internalization into tumours. *Br. J. Cancer*, **62** (suppl. X), 1–5.

MICHALIDES R, KWA B, SPRINGALL D, VAN ZANDWIJK N, KOOPMAN J, HILKENS J AND MOOI W. (1994). NCAM and lung cancer. *Int. J. Cancer*, **8** (suppl.), 34–37.

MOOLENAAR CEC, MULLER EJ, SCHOL DJ, FIGDOR CG, BOCK E, BITTER-SUERMANN D AND MICHALIDES RJAM. (1990). Expression of neural cell adhesion molecule related sialoglycoprotein in small cell lung cancer and neuroblastoma cell lines H69 and CHP-212. *Cancer Res.*, **50**, 1102–1106.

NELP WB, GRIEP RG, SALK D, ABRAMS P, SUPPERS V AND HANLEY M. (1990). Successful staging of small cell lung cancer (SCLC) with monoclonal antibody. *Eur. J. Nucl. Med.*, **16**, S199.

PRESS OW, FARR AG, BORROZ KI, ANDERSON SK AND MARTIN PJ. (1989). Endocytosis and degradation of monoclonal antibodies targeting human B-cell malignancies. *Cancer Res.*, **49**, 4906–4912.

RYGAARD K, MØLLER C, BOCK E AND SPANG-THOMSEN M. (1992). Expression of cadherin and NCAM in human small cell lung cancer cell lines and xenografts. *Br. J. Cancer*, **65**, 573–577.

SCHOL DJ, MOOI WJ, VAN DER GUGTEN AA, WAGENAAR SJSc AND HILGERS J. (1988). Monoclonal antibody 123C3, identifying small cell carninoma phenotype in lung tumors, recognizes mainly, but not exclusively, endocrine and neuron-supporting normal tissues. *Int. J. Cancer*, **43** (suppl.2), 34–40.

THE TH AND FELTKAMP TEW. (1970). Conjugation of fluorescein isothiocyanate to antibodies. *Immunology*, **18**, 865–881.

WAIBEL R, MANNHART M, O'HARA CJ, BROCKLEHURST C, ZANGEMEISTER-WITTKE U, SCHENKER T, LEHMANN HP, WEBER E AND STAHEL RA. (1993). Monoclonal antibody SEN7 recognizes a new epitope on the neural cell adhesion molecule present on small cell lung cancer but not on lymphocytes. *Cancer Res.*, **53**, 2840–2845.

YESNER R. (1985). Classification of lung cancer histology. *N. Engl. J. Med.*, **312**, 652–653.